# LONG-TERM CARE INSURANCE AND OVERLOOKED LONG-TERM CARE EXPENSES AMONG OLDER ADULTS WITH COGNITIVE IMPAIRMENTS

**DOI:** 10.1093/geroni/igae098.0461

**Published:** 2024-12-31

**Authors:** Chengxu Long, Wei Yang, Karen Glaser

**Affiliations:** King’s College London, London, England, United Kingdom; King’s College London, London, England, United Kingdom; King’s College London, London, England, United Kingdom

## Abstract

We investigate catastrophic health and long-term care expenditures (CHLTCE) among older adults with cognitive impairments in China and the role played by long-term care insurance (LTCI). Drawing on data from the 2013, 2015, 2018, and 2020 waves of the China Health and Retirement Longitudinal Study, we exploit the rollout of LTCI pilots across different cities and employ a difference-in-differences approach. Our findings indicate that a significant proportion of CHLTCE among older adults with cognitive impairment is attributed to long-term care costs (i.e., both formal and informal). After considering long-term care expenditure, both the incidence and intensity of CHLTCE increase significantly compared to when only healthcare expenditures are taken into account. Our research underscores the importance of considering long-term care costs when examining catastrophic expenditures due to both health and long-term care expenditures among older adults with cognitive impairment. We also find that the implementation of LTCI reduces both the incidence and the intensity of CHLTCE among older adults with cognitive impairment. Moreover, this effect is more pronounced among individuals with more severe cognitive impairment and those who live alone. These findings suggest that the introduction of LTCI, may, to some extent, alleviate CHLTCE among older adults with cognitive impairments. Our findings support the extension of the LTCI pilot as well as the broadening of the insurance’s scope to ensure adequate protection for older adults with cognitive impairments.

